# Intra-osseous plasma rich in growth factors enhances cartilage and subchondral bone regeneration in rabbits with acute full thickness chondral defects: Histological assessment

**DOI:** 10.3389/fvets.2023.1131666

**Published:** 2023-03-29

**Authors:** Marta Torres-Torrillas, Elena Damia, Ayla del Romero, Pau Pelaez, Laura Miguel-Pastor, Deborah Chicharro, José M. Carrillo, Mónica Rubio, Joaquín J. Sopena

**Affiliations:** ^1^Bioregenerative Medicine and Applied Surgery Research Group, Department of Animal Medicine and Surgery, CEU Cardenal Herrera University, CEU Universities, Valencia, Spain; ^2^García Cugat Foundation CEU-UCH Chair of Medicine and Regenerative Surgery, CEU Cardenal Herrera University, CEU Universities, Valencia, Spain

**Keywords:** platelet rich plasma, growth factors, osteoarthritis, chondral defect, articular cartilage, histology, bioregenerative therapies

## Abstract

**Background:**

Intra-articular (IA) combined with intra-osseous (IO) infiltration of plasma rich in growth factors (PRGF) have been proposed as an alternative approach to treat patients with severe osteoarthritis (OA) and subchondral bone damage. The aim of the study is to evaluate the efficacy of IO injections of PRGF to treat acute full depth chondral lesion in a rabbit model by using two histological validated scales (OARSI and ICRS II).

**Methodology:**

A total of 40 rabbits were included in the study. A full depth chondral defect was created in the medial femoral condyle and then animals were divided into 2 groups depending on the IO treatment injected on surgery day: control group (IA injection of PRGF and IO injection of saline) and treatment group (IA combined with IO injection of PRGF). Animals were euthanized 56 and 84 days after surgery and the condyles were processed for posterior histological evaluation.

**Results:**

Better scores were obtained in treatment group in both scoring systems at 56- and 84-days follow-up than in control group. Additionally, longer-term histological benefits have been obtained in the treatment group.

**Conclusions:**

The results suggests that IO infiltration of PRGF enhances cartilage and subchondral bone healing more than the IA-only PRGF infiltration and provides longer-lasting beneficial effects.

## 1. Introduction

Osteoarthritis (OA) developing from age-related wear and tear, chronic diseases or traumatic injuries can lead to progressive cartilage degeneration and damage to osteochondral junction and subchondral bone (SB) (1). Pathological changes observed in OA joints include progressive destruction of articular cartilage (AC), thickening of the SB, swelling of the synovium, degeneration of intra-articular (IA) structures such as ligaments and menisci, osteophytes formation, and hypertrophy of the joint capsule (2). The relevance of the pathology lies in its high prevalence and the continuous pain suffered by the patient, limiting its quality of life. OA affects over 20% of canine population (3, 4), 90% of cats over 12 years of age (5, 6), and more than 50% of horses older than 15 years (7–9), which makes OA the most diagnosed joint disease in companion animals.

Although a great variety of treatment, such as anti-inflammatory drugs and analgesics, have been used to reduce pain and improve functionality, so far these treatments can only relieve symptoms and do not regenerate the damaged tissue (10). AC lacks blood vessels, giving it a very limited natural regenerative potential, so full thickness chondral defects generally require clinical intervention to enable lesion healing (11). During the last years AC repair through tissue engineering has become a promising alternative, and IA infiltrations of platelet rich plasma (PRP) have been widely used as an effective biological approach to improve AC regeneration due to its chondro-inductive properties and abundant pool of growth factors (GF) (12, 13).

PRP is composed of different GF and proteins such as fibrinogen, vitronectin and fibronectin that play a key role during AC repair (14). Several GF have demonstrated to be crucial in cartilage regeneration including transforming growth factor β, involved in matrix production, cell proliferation and chondrogenic differentiation of mesenchymal stromal cells (MSCs) implicated in collagen type II expression; insulin-like growth factor-1, which modulates chondrogenesis and induce MSCs proliferation; platelet-derived growth factor (PDGF) that stimulates angiogenesis and proteoglycan production; and fibroblast growth factor-2, which promotes chondrocyte and MSCs proliferation (15).

Despite the positive outcomes reported in several studies, this cell free therapy still has some limitations, such as the differences between PRP obtention protocols and thus differences in composition and platelet concentration. Plasma rich in growth factors (PRGF) (PRGF^®^-Endoret^®^, BTI Biotechnology Institute, Spain) is a specific technology that allows obtaining, in a single centrifugation process, an autologous PRP preparation with a platelet concentration 2.5 times higher than that of whole blood, and with absence of white and red blood cells (16, 17). This technology reduces proinflammatory activity, and several studies have assessed its clinical use in orthopedics and other medical fields (18).

Another limitation of this bioregenerative therapy is due its administration route. PRP is commonly administrated via IA injections, which have shown to be effective in patients with mild or moderate OA (19, 20). However, some authors have recently described its limitation in patients with severe degrees of OA, when the SB is affected since, PRP cannot reach the deeper layers of the AC or the SB (21, 22).

The SB is the bony tissue lying beneath the calcified zone of the AC and includes both, the cortical plate and the subchondral trabecular bone. The subchondral bone plate is a thin layer of cortical bone underlying the calcified cartilage that provides mechanical strength to support the AC. Additionally, in the cortical plate there are canals that allow the exchange of nutrients and molecules between the SB and the AC (23). The subchondral trabecular bone lies underneath the subchondral bone plate, and it is more porous and metabolically active (24). The osteochondral unit in the joint consists of AC, calcified cartilage, and SB; and it is responsible for transfer loads during joint movement. In OA, the integrity of the osteochondral unit is breached, and the cross-talk between cartilage and SB is increased. SB undergoes drastic changes in composition and structural organization that negatively affects the overlaying AC (24). In early stages of the disease, bone loss is found, followed by increased trabecular thickness and, in severe stages, cancellous bone collapse can be present (25). These structural changes in the SB lead to the activation of proinflammatory cytokines, both in the SB and the AC, thus perpetuating OA (26). Increasing evidence points to SB as the primary tissue responsible for pain and chronic inflammation, which may be the main driver of OA (27–29). Moreover, the SB is an important element in maintaining joint homeostasis and it is directly communicated with the AC, so any change in the SB microenvironment can affect cartilage metabolism (30, 31).

Combining intra-osseous (IO) and IA PRP injections to target SB can provide a more complete treatment by stimulating biological processes that lead to a homeostatic environment in the whole joint, which also influences the behavior of MSCs that coordinate SB and AC remodeling (32, 33). Various research groups have already showed that the IO approach is safe and provides longer-term functional and clinical benefits compared to IA injections of PRP alone (34–37). Moreover, our research team has previously investigated the efficacy of the IO infiltration technique in acute chondral lesions in rabbits, and better macroscopical and serum biomarkers results were obtained in animals in which IA PRP was combined with IO infiltration (38, 39).

The present study hypothesizes that IA together with IO infiltration of PRGF could improve the regenerative potential of IA PRGF infiltration alone. With that basis, the aim of the study is to histologically assess the effectiveness of IO PRGF in a model of full depth chondral defects in rabbits by using two validated scales (40, 41).

## 2. Materials and methods

The Ethics Committee of Animal Welfare (CEEA) of the University CEU Cardenal Herrera of Valencia (Spain) approved the study with the following approval code: 2019/VSC/PEA/0153, in accordance with the Spanish policy for Animal Protection (RD118/2021), which conforms with the European Union Directive 2010/63/UE.

### 2.1. Animals

A total of 40 female New Zealand rabbits (6 months old), 20 animals per treatment group (control or treatment) with a mean weight of 4.24 kg were used to complete a prospective randomized experimental study. Animals were allowed to have food and water ad libitum and were housed in individual big cages without movement restriction. An acclimatation period of 15 days was established at the beginning of the study to allow rabbits adaptation. Inclusion criterion consisted of a normal physical examination and a normal hematology and serum biochemical analysis. These tests were completed 10 days prior to beginning the experiment and all results were considered normal reference values for the species.

During all the study time, rabbits were examined daily to detect signs of pain, infection, and weight loss. The rabbit Grimace Scale was performed twice a day during the first 7 days after the surgery and once a day during the rest of the study. If a score equal or >4 was obtained, buprenorphine 0.1 mg/kg SC q8h (Bupaq^®^, Richter Pharma AG, Austria) was given as a rescue analgesia. If any animal showed critical aggravation of their functional or physical condition, they were excluded from the study. Only one animal was excluded due to a severe infection in the surgical area.

At the end of the study, rabbits were euthanized in agreement with Spanish Policy for Animal Protection (RD118/2021).

### 2.2. Study groups

Depending on the IO treatment received, animals were divided into 2 groups of 20 animals each:

- Control group (CT): IA infiltration with PRGF combined with IO infiltration with sterile saline solution.
- Treatment group (TRT): IA infiltration with PRGF combined with IO infiltration with PRGF.

### 2.3. Plasma rich in growth factors

**Figure 1:**
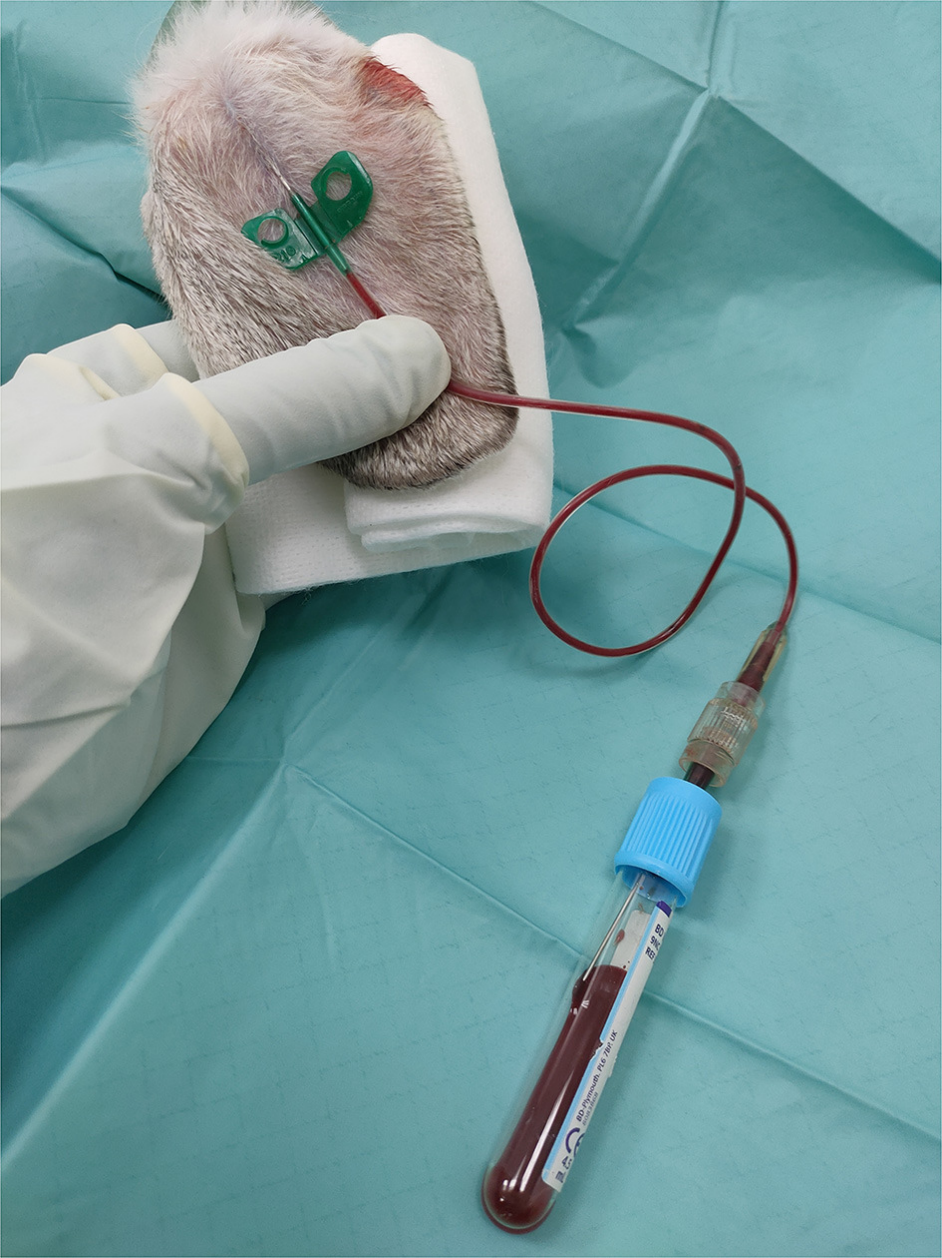
Blood extraction from the auricular artery for PRGF obtention. A 21G Venofix connected to a 21G hypodermic needle by a male-male luer-lock connector was used for this purpose. The blood was collected in vacutainer sodium citrate 3.8% tubes.

Rabbits were sedated with IM dexmedetomidine (0.05 mg/kg; Dexdomitor^®^, Esteve, Spain), IM ketamine (10 mg/kg; Imalgene^®^, Merial, Spain), and IM morphine (1 mg/kg; B-Braun^®^, Germany). After sedation, a total of 15 mL of blood were collected from the auricular artery of each rabbit under sterile conditions in vacutainer sodium citrate 3.8% tubes (BD Vacutainer^®^ 9NC, New Jersey, USA) (Figure 1). PRGF^®^-Endoret^®^ technology was used to obtain an autologous preparation of PRP. The blood tubes were centrifugated at 460 g for 8 min (PRGF^®^ System III, Biotechnology Institute^®^, Álava, Spain) to separate the different blood phases. A sterile fractionation tube was used to collect PRGF with sterile pipettes. Just before IA or IO infiltration, 10% calcium chloride was added to the PRGF (50 μl/ml of PRGF) to activate platelets for GF release.

### 2.4. Articular cartilage defect model

Following sedation and anesthesia induction with sevoflurane (Sevoflo^®^, Esteve, Spain), a full thickness chondral defect was created in the rabbits' knees. Both lower limbs were shaved and trimmed with an antiseptic solution prior to surgical intervention.

The rabbit's knee was kept in complete flexion to perform a skin incision 10 mm long over the margin of the medial femoral condyle, followed by an incision of the fascia and joint capsule. The medial femoral condyle was then exposed, and the loading area was identified. A full thickness chondral defect, 4 mm in diameter and 5 mm in depth was then created with a drill bit and with the help of a drilling guide. The blood that oozed out after the creation of the defect has cleared with sterile saline solution and dried with sterile gauzes. Both knees were subjected to an identical surgical procedure. After creating the defect, simple stitches were used to close the joint capsule, fascia and skin using 3/0 polyglyconate (Novosyn^®^ Quick, B-Braun, Germany).

Following surgery all animals were treated with anti-inflammatory drugs (meloxicam 0.3 mg/kg SC q24h; (Metacam^®^, Boehringer Ingelheim, Spain) and antibiotic (enrofloxacin 10 mg/kg SC q24h; Ganadexil^®^, Invesa, Spain) for a 7-day period. Rabbits were monitored daily, if any animal showed radical worsening in their physical or functional condition, such as severe local infection or severe traumatic lesions, they were excluded from the study.

### 2.5. Intra-articular and intra-osseous infiltration

**Figure 2:**
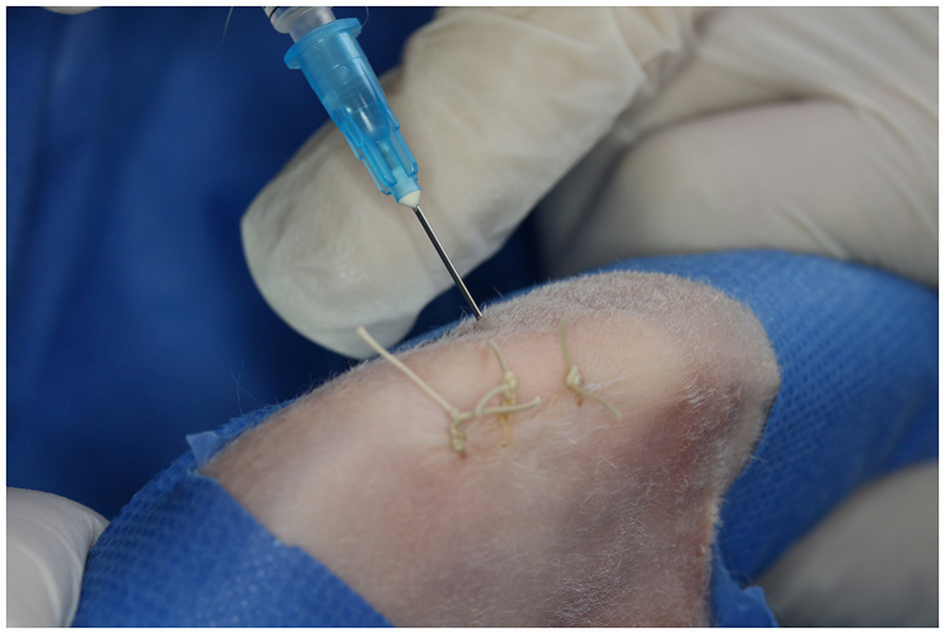
Intra-articular infiltration with plasma rich in growth factors. A 22-G needle was inserted laterally to the patellar tendon and 0.25 mL of plasma rich in growth factors were IA injected.

**Figure 3:**
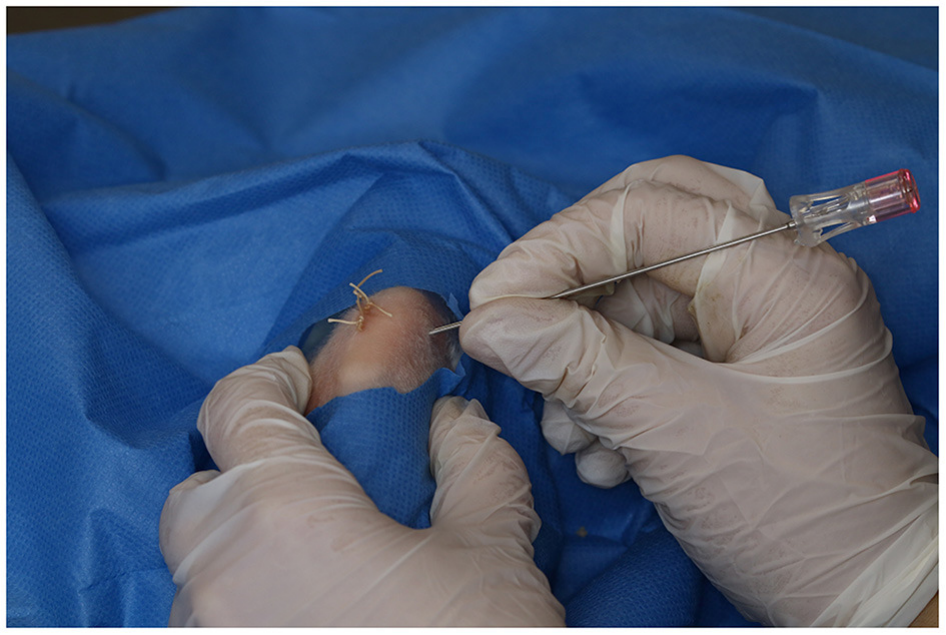
Intra-osseous infiltration of plasma rich in growth factors. An 18-G spinal needle was inserted perpendicular to the femur in the lateral supracondylar region with gentle rotation movements.

With the knee in complete flexion, a 22-G needle was inserted laterally to the patellar tendon and 0.25 mL of PRGF were IA injected in all animals regardless of the treatment group (Figure 2). Finally, with the limb in a relaxed position, the IO infiltration was performed with an 18-G spinal needle inserted perpendicular to the femur in the lateral supracondylar region with gentle rotation movements, and 0.5 mL of sterile saline solution or PRGF depending on the treatment group (CT or TRT respectively) were injected (Figure 3). A mean time of 7.21 min elapsed from defect creation to IA and IO infiltration.

### 2.6. Animals' euthanasia

Animals were euthanized in compliance with Spanish Policy for Animal Protection (RD118/2021). Rabbits were sedated with dexmedetomidine (0.05 mg/kg IM; Dexdomitor^®^, Esteve, Spain) and ketamine (10 mg/kg IM; Imalgene^®^, Merial, Spain), and thereafter and IV injection of pentobarbital sodium (150 mg/kg; Dolethal^®^, Vetoquinol, France) in the marginal ear vain was given.

A total of 19 rabbits were euthanized 56- days following the infiltration (9 animals from the CT group and 10 animals from the TRT group). The rest of the animals (10 animals from the CT group and 10 animals from the TRT group) were euthanized at 84- days follow up.

### 2.7. Tissue processing, sectioning, and staining

After animals were euthanized both femurs were excised and fixed in neutral buffered formaldehyde 4% for 15 days and then decalcified in bisodic EDTA in acid buffer (Osteodec^®^, Bio-Optica, Milano, Italy) for 5 weeks. Every week the samples were re-fixed in neutral buffered formaldehyde 4% for 48 h. Then, the decalcified tissue was dehydrated for 50 h in a tissue processor (Leica biosystems^®^, Amsterdam, Netherlands) using a series of 70–100% ethanol, cleared in xylene and embedded in paraffin. Paraffin-embedded sections were cut at 5 μm on a microtome in parasagittal plane and six sections per condyle were evaluated. The sections were stained with Weigert's Hematoxyln and Safranin-O Fast Green and the joint degeneration was evaluated using the OARSI histological score proposed for rabbits (41), and an adaptation of the ICRS II scoring system (40). A photomicroscope and an attached digital camera were used to digitalize the stained sections, which were evaluated by a blinded pathologist.

The OARSI histological assessment of articular cartilage changes in the rabbit model of OA is described in Table 1 (41), and the adapted ICRS II scoring system is explained in Table 2 (40).

**Table 1:** OARSI Histological assessment of articular cartilage changes in rabbit model of OA.

**Parameter**	**Score**	**Description**
Safranin-O staining	0	Uniform staining throughout articular cartilage
	1	Loss of staining in superficial zone of hyaline cartilage < 50% of the length of the condyle
	2	Loss of staining in superficial zone of hyaline cartilage ≥ 50% of the length of the condyle
	3	Loss of staining in the upper 2/3's of hyaline cartilage < 50% of the length of the condyle
	4	Loss of staining in the upper 2/3's of hyaline cartilage ≥ 50% of the length of the condyle
	5	Loss of staining in all the hyaline cartilage < 50% of the length of the condyle
	6	Loss of staining in all the hyaline cartilage ≥ 50% of the length of the condyle
Structure	0	Normal
	1	Surface irregularities
	2	Fissures in < 50% of the surface
	3	Fissures in ≥ 50% of the surface
	4	Erosion 1/3 hyaline cartilage < 50% of the surface
	5	Erosion 1/3 hyaline cartilage ≥ 50% of the surface
	6	Erosion 2/3 hyaline cartilage < 50% of the surface
	7	Erosion 2/3 hyaline cartilage ≥ 50% of the surface
	8	Full depth erosion hyaline cartilage < 50% of the surface
	9	Full depth erosion hyaline cartilage ≥ 50% of the surface
	10	Full depth erosion hyaline and calcified cartilage to the subchondral bone < 50% of the surface
	11	Full depth erosion hyaline and calcified cartilage to the subchondral bone ≥ 50% of the surface
Chondrocyte Density	0	No decrease in cells
	1	Focal decrease in cells
	2	Multifocal decrease in cells
	3	Multifocal confluent decrease in cells
	4	Diffuse decrease in cells
Cluster Formation	0	Normal
	1	< 4 clusters
	2	≥ clusters but < 8 clusters
	3	≥ 8 clusters

Obtained from The OARSI histopathology initiative. For the purposes of the OARSI histological score for rabbits, articular cartilage is divided into four zones: superficial zone (upper 1/3 of hyaline cartilage), middle zone (middle 1/3 of hyaline cartilage), deep zone (deep 1/3 of hyaline cartilage), and the calcified cartilage. The scale ranges from 0 to 24 points, being 24 the worst possible result (41).

**Table 2:** ICRS II parameters and scores description.

**Parameter**	**Score**	**Description**
Matrix staining	0%	No staining
	10%	Full metachromasia
Cell morphology	0%	No round/ oval cells
	100%	Mostly round/oval cells
Chondrocyte clustering	0%	Present
	100%	Absent
Surface architecture	0%	Delamination, major irregularity
	100%	Smooth surface
Basal integration	0%	No integration
	100%	Complete integration
Formation of a tidemark	0%	No calcification front
	100%	Tidemark
Subchondral bone abnormalities	0%	Abnormal
	100%	Normal marrow
Inflammation	0%	Present
	100%	Absent
Abnormal calcification	0%	Present
	100%	Absent
Vascularization	0%	Present
	100%	Absent
Superficial assessment	0%	Total loss or complete disruption
	100%	Resembles intact articular cartilage
Mid/deep zone assessment	0%	Fibrous tissue
	100%	Normal hyaline cartilage
Overall assessment	0%	Bad (fibrous tissue)
	100%	Good (hyaline cartilage)

The criteria identified to represent the ICRS II scoring system comprise 13 parameters. Each criterion should be evaluated based on the visual analog scale and graded from 0 (worst) to 100 (best) (41).

### 2.8. Statistical analysis

A power analysis consistent with results published in previous research, in which only IA PRP was infiltrated (42), was used to calculate the sample size. An alpha level of 0.02 and a power of 80% were established.

The data were processed using the SPSS 20.0 program for Windows (SPSS^®^Inc., Chicago, USA). A descriptive study of the mean and standard deviation was performed for each variable. Mann-Whitney test was used to compare the variables. A *p* < 0.05 was considered significant.

## 3. Results

A total of 39 rabbits completed the study (19 in the CT group and 20 in the TRT group). One rabbit was excluded from the study due to a severe multidrug resistant infection in the surgical area.

Regarding the platelet concentration in whole blood, a mean value of 439,984 x 10^3^ ± 180,179 x 10^3^/ μl was obtained. However, after blood centrifugation, the mean platelet concentration within the PRGF fraction was 1023 × 10^3^ ± 341,183 × 10^3^/ μl.

### 3.1. OARSI histological score

Significant differences between the CT group and the TRT group were demonstrated for each studied parameter at both study times.

In the group of rabbits sacrifice at 56-days follow-up, significantly higher scores were observed in the CT group for criterion safranin-O staining (*p* < 0.001), AC structure (*p* < 0.001), chondrocyte density (*p* < 0.001) and cluster formation (*p* = 0.001) than in the TRT group. Accordingly, the CT group showed a significantly greater total score than the TRT group (*p* < 0.001). The mean and standard deviation for each parameter are described in Table 3.

**Table 3:** OARSI score means, standard deviation and *p*-values.

	**56- days**			**84- days**		
**OARSI score**	**CT**	**TRT**	***P*** **-value**	**CT**	**TRT**	***P*** **-value**
Safranin-O Staining	3.80 ± 1.15	1.88 ± 0.50	< 0.001	3.29 ± 1.07	0.94 ± 0.85	< 0.001
Articular Cartilage Structure	6.00 ± 1.41	2.13 ± 0.81	< 0.001	4.71 ± 1.73	1.13 ± 0.81	< 0.001
Chondrocyte Density	3.40 ± 0.99	1.31 ± 0.87	0.001	2.79 ± 0.96	1.13 ± 0.62	< 0.001
Cluster Formation	2.40 ± 0.51	1.56 ± 0.51	< 0.001	2.29 ± 0.83	0.94 ± 0.772	< 0.001
Total score	15.60 ± 2.41	6.88 ± 1.63	< 0.001	13.07 ± 2.73	4.13 ± 2.16	< 0.001

CT, Control group; TRT, treatment group.

Similar results were shown in rabbits 84-days after infiltration. Significantly higher scores were reported in the CT group than in the TRT group for the following OARSI scale parameters: safranin-O staining (*p* < 0.001), AC structure (*p* < 0.001), chondrocyte density (*p* < 0.001) and cluster formation (*p*-value < 0.001). Hence, the TRT group showed significantly lower total score than the CT group (*p* < 0.001). The mean and standard deviation for each criterion are described in Table 3.

Moreover, the CT group showed significantly higher scores at-56 days follow-up than at 84-days follow-up for the parameter AC structure (*p* = 0.037), while no significant differences were reported for the criterion: safranin-O staining (*p* = 0.2239), chondrocyte density (*p* = 0.103), cluster formation (*p* = 0.654) and total score (*p* = 0.062).

On the other hand, significantly higher scores were showed in the TRT group 56-days following infiltration than 84-days after the infiltration for the following parameters: safranin-O staining (*p* < 0.001), AC structure (*p* = 0.001), cluster formation (*p* = 0.011) and total score (*p* < 0.001) (Figure 1). No significant differences between sacrifice time were reported in the TRT group for the criteria chondrocyte density (*p* = 0.489).

**Figure 4:**
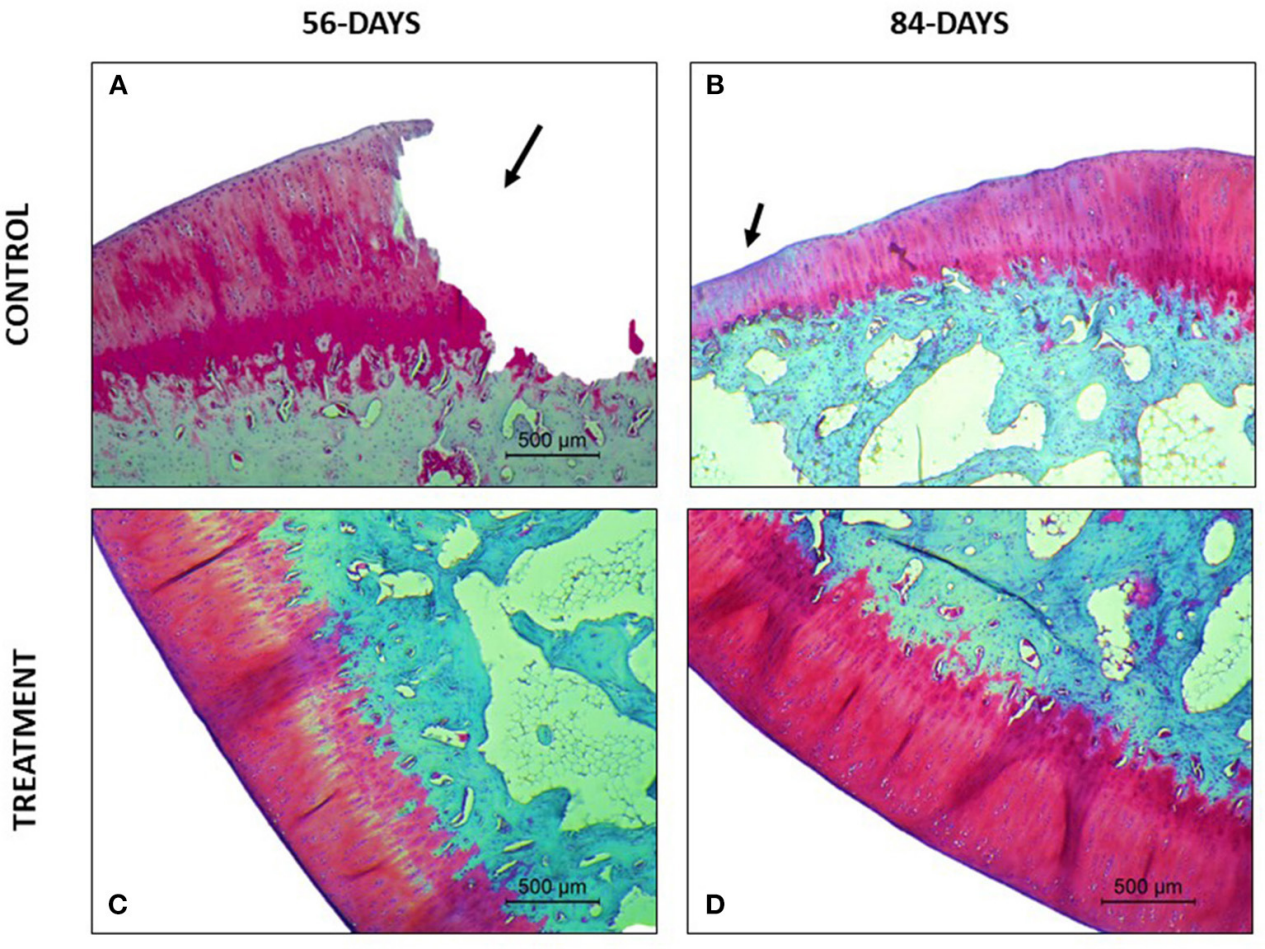
Safranin O- Fast green stained sections. **(A)** Complete loss of superficial, mid and deep zones of the articular cartilage. **(B)** Loss of articular cartilage thickness in the defect area. Loss of safranin-o staining indicating a loss of proteoglycans. **(C)** Good proteoglycan distribution with some areas with loss of staining. **(D)** Full extracellular matrix staining.

**Figure 5:**
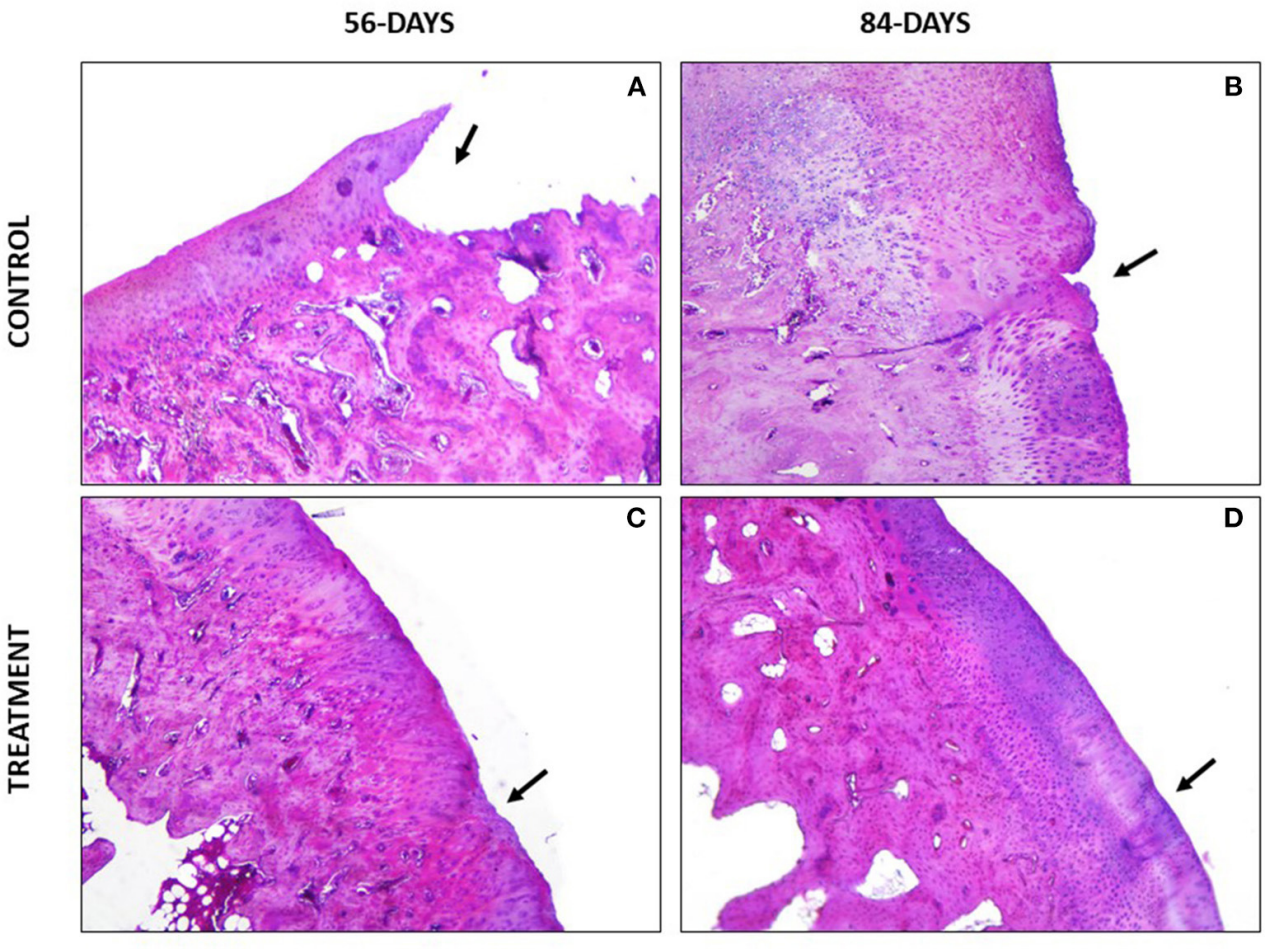
Hematoxylin-Eosin stained sections. **(A)** Loss of superficial, mid and deep layers of the articular cartilage. **(B)** Clefts in the superficial zone and structural disorganization. **(C)** Small superficial erosion. **(D)** Good superficial integration with small irregularities.

**Figure 6:**
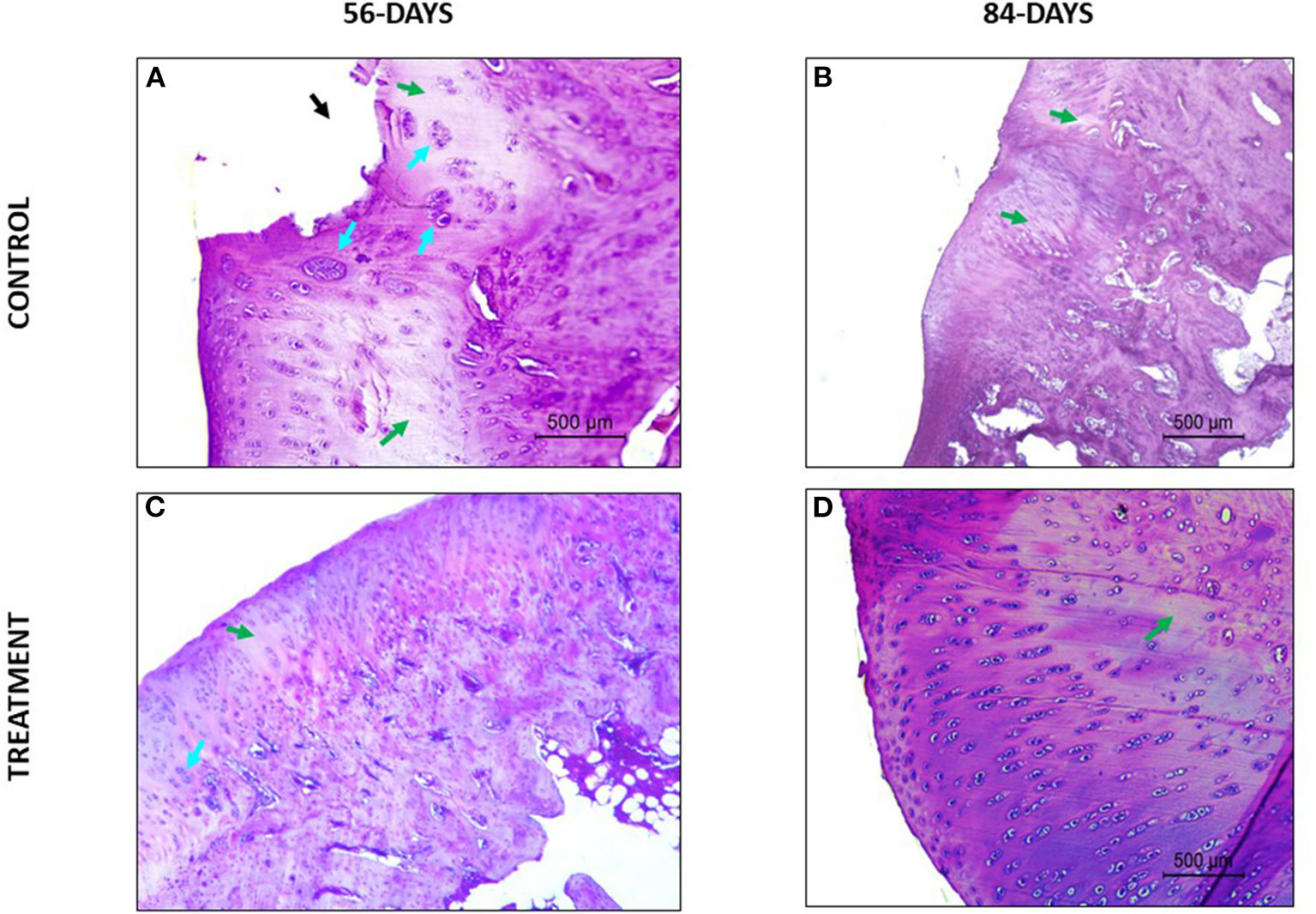
Hematoxylin-Eosin stained sections. Green arrows point areas of chondrocyte density decrease and blue arrows point chondrocytes clusters. **(A)** Lack of superficial and mid zones of the articular cartilage (black arrow), chondrocyte clustering and multifocal and confluent eosinophilic areas with lack of chondrocytes. **(B)** Eosinophilic areas with diminished chondrocyte density and structural disorganization. **(C)** Focal areas of hypocellullarity and some chondrocytes aggrupation. **(D)** Good chondrocytes organization and morphology (oval and rounded cells organized in columns) without cluster presence and basophilic extracellular matrix.

Regarding the parameter safranin-o staining, a loss of staining in the superficial and mid/ deep zones was reported in the CT group at both study times, thus meaning a lack in proteoglycan content; however, in the TRT group the staining was uniform throughout AC (Figure 4). Moreover, for the parameter structure, in the TRT group some small fissures and surface irregularities were reported at both studied times; nonetheless, in the CT group full depth erosions of the hyaline cartilage were reported at 56 days follow up, and erosions of 2/3 of hyaline cartilage were reported 84 days following infiltration (Figure 5). With regards to chondrocyte density, multifocal areas of chondrocytes decrease were identified in the condyles of CT rabbits at both study times, while for TRT animals focal decrease was reported (Figure 6). Finally, regarding cluster formation, the CT group showed an elevated presence of chondrocytes clusters both at 56- and 84-days follow-up; however, a lower presence of clusters was reported for the TRT group (Figure 6).

### 3.2. ICRS II histological score

Significant differences were demonstrated between groups at both study times for each variable of the ICRS II Histological Score.

In the rabbits euthanized 56-days after the infiltration, significantly higher scores were reported in the TRT group than in the CT group for the following studied parameters: matrix staining (*p* < 0.001), cell morphology (*p* < 0.001), chondrocyte clustering (*p*-value 0.01), surface architecture (*p* < 0.001), basal integration (*p*-value 0.001), formation of a tidemark (*p* < 0.001), SB abnormalities and marrow fibrosis (*p* < 0.001), inflammation (*p* < 0.001), abnormal calcification (*p* = 0.001), surface assessment (*p* < 0.001), mid/deep zone assessment (*p* < 0.001) and overall assessment (*p* < 0.001). Contrarily, significantly lower score was showed in the TRT group for the criteria vascularization (*p* = 0.001) than in the CT group. The mean and standard deviation for each parameter are described in Table 4.

**Table 4:** ICRS II score mean and standard deviation at 56 and 84 days follow-up.

	**56-days**			**84-days**		
**ICRS II score**	**CT**	**TRT**	***P*** **-value**	**CT**	**TRT**	***P*** **-value**
Matrix staining	52.50 ± 14.83	77.81 ± 11.10	< 0.001	47.14 ± 12.67	84.69 ± 10.56	< 0.001
Cell morphology	40.00 ± 9.66	71.88 ± 10.47	< 0.001	53.57 ± 13.36	85.00 ± 8.94	< 0.001
Chondrocyte clustering	45.63 ± 13.65	61.88 ± 16.82	0.01	47.14 ± 18.16	85.00 ± 10.94	< 0.001
Surface architecture	45.63 ± 14.13	68.13 ± 9.11	< 0.001	56.07 ± 11.13	78.75 ± 8.66	< 0.001
Basal integration	28.13 ± 21.05	74.38 ± 13.65	0.001	61.43 ± 10.99	81.25 ± 10.88	< 0.001
Formation of a tidemark	23.75 ± 8.85	67.50 ± 13.43	< 0.001	35.08 ± 14.14	80.06 ± 11.55	< 0.001
Subchondral bone abnormalities and marrow fibrosis	43.75 ± 9.57	82.50 ± 11.26	< 0.001	60.03 ±10.38	81.88 ± 9.81	< 0.001
Inflammation	65.00 ± 16.73	86.25 ± 8.06	< 0.001	58.57 ± 28.52	94.06 ± 7.58	< 0.001
Abnormal calcification	67.50 ± 16.93	87.50 ± 10.00	< 0.001	71.43 ± 19.16	98.75 ± 3.42	< 0.001
Vascularization	60.63 ± 16.92	50.31 ± 6.45	0.001	67.14 ± 13.82	78.44 ± 8.11	0.004
Surface assessment	42.81 ± 14.60	69.69 ± 6.48	< 0.001	53.57 ± 12.77	80.09 ± 7.30	< 0.001
Mid/deep zone assessment	34.06 ± 4.91	66.56 ± 11.06	< 0.001	52.86 ± 10.08	79.38 ± 6.81	< 0.001
Overall assessment	38.44 ± 5.07	71.56 ± 11.21	< 0.001	53.57 ± 10.08	81.88 ± 8.34	< 0.001
Mean score	43.08 ± 6.56	72.28 ± 4.81	< 0.001	53.10 ± 9.31	83.77 ± 4.06	< 0.001

CT, Control group; TRT, Treatment group.

Furthermore, in rabbits euthanized 84-days following the infiltration, significantly higher scores were reported in the TRT group than in the CT group for all the criterion included in the ICRS II score: matrix staining (*p* < 0.001), cell morphology (*p* < 0.001), chondrocyte clustering (*p* < 0.001), surface architecture (*p* < 0.001), basal integration (*p* < 0.001), formation of a tidemark (*p* < 0.001), SB abnormalities and marrow fibrosis (*p* < 0.001), inflammation (*p* < 0.001), abnormal calcification (*p* = 0.001), vascularization (*p* = 0.004), surface assessment (*p* < 0.001), mid/deep zone assessment (*p* < 0.001) and overall assessment (*p* < 0.001). The mean and standard deviation of each parameter are described in Table 4.

Moreover, the CT group showed no statistical differences between the obtained score at 56-days follow-up and at 84 days follow-up for the following criterion: matrix staining (*p* = 0.30), chondrocyte clustering (*p* = 0.79), SB architecture (*p* = 0.06), inflammation (*p* = 0.45), abnormal calcification (*p* = 0.22) and vascularization (*p* = 0.26). However, statistical differences between different the sacrifice points were reported for the following criterion: cell morphology (*p* = 0.003), surface architecture (*p* = 0.001), basal integration (*p* < 0.001), formation of a tidemark (*p* = 0.014), surface assessment (*p* = 0.001), mid/deep zone assessment (*p* < 0.001) and overall assessment (*p* < 0.001).

Regarding TRT group, no statistical differences between the results obtained 56-days following the infiltration and the ones obtained at 84-days were reported for the following parameters: matrix staining (*p* = 0.083), basal integration (*p* = 0.126), formation of a tidemark (*p* = 0.095) and SB abnormalities (0.868). On the other hand, statistical differences between the two sacrifice times were reported for the following criterion: cell morphology (*p* < 0.001), chondrocytes clustering (*p* < 0.001), surface architecture (*p* = 0.002), inflammation (*p* = 0.008), abnormal calcification (*p* < 0.001), vascularization (*p* < 0.001), surface assessment (*p* < 0.001), mid/deep zone assessment (*p* < 0.001) and overall assessment (*p* = 0.006).

**Figure 7:**
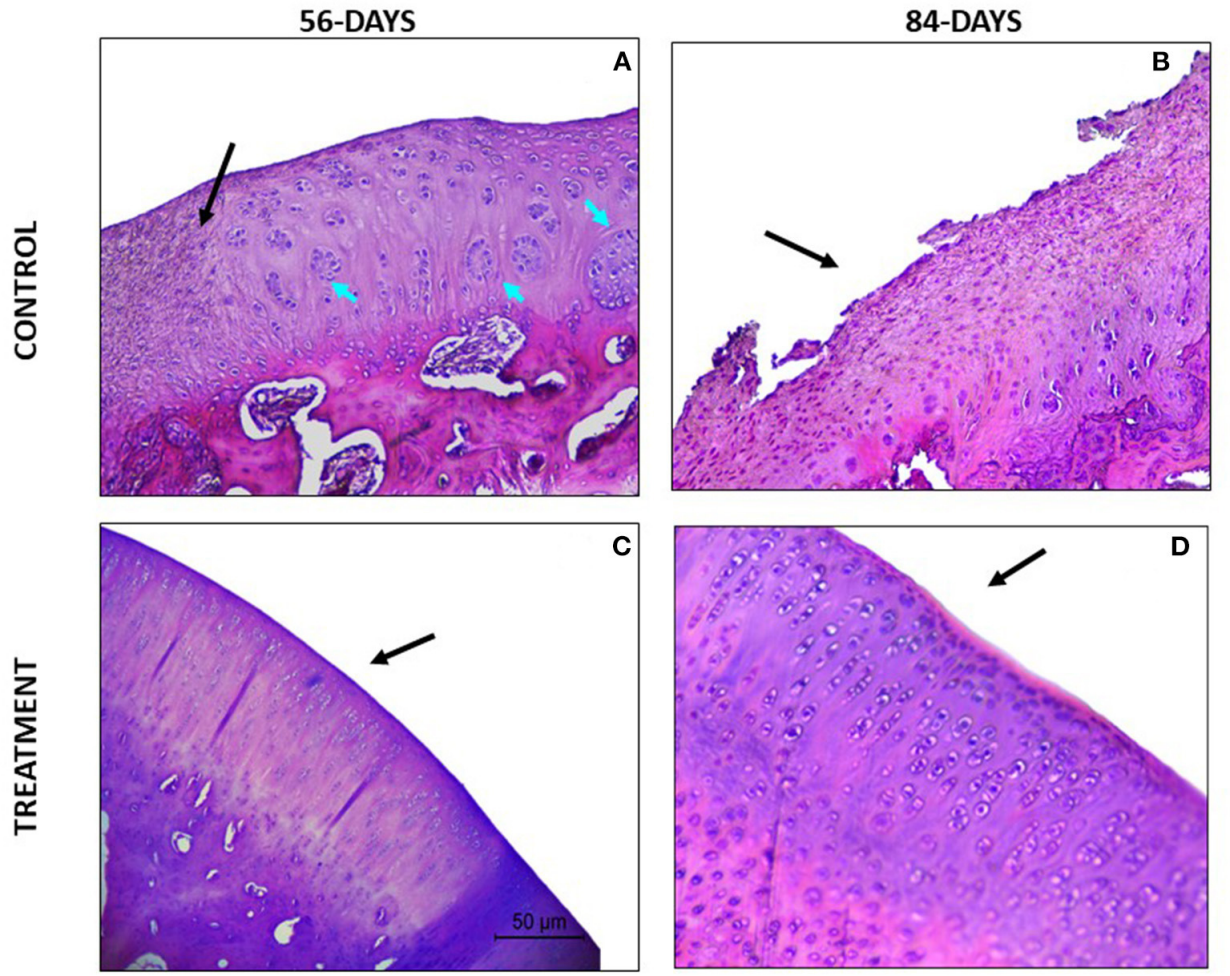
Hematoxylin-Eosin stained sections. **(A)** The defect area is being filled with fibrocartilage-like tissue (black arrow). Additionally, big chondrocytes clusters are present (blue arrows). **(B)** Defect filled with fibrocartilage-like tissue. **(C)** Good regeneration tissue, hyaline like appearance. Some areas with low chondrocyte density can be identified. **(D)** High chondrocyte density, all of them forming columns. The tissue is comparable to healthy hyaline cartilage.

For the parameter matrix staining, a lack of safranin- O staining was present in the CT group (Figure 4) and, when hematoxylin-eosin stained the repaired cartilage in the CT group showed to be more eosinophilic than the repaired cartilage in the TRT group at both follow-up times. With regards to cell morphology, the CT group showed a lower presence of rounded or oval cells, while in the TRT group most cells were rounded. Moreover, an increased presence of chondrocytes clusters was reported in the CT group compared to TRT group (Figure 6). Regarding the criteria surface architecture, surface assessment and mid/deep zone assessment, in the TRT group small surface fissures with mostly normal organization of chondrocytes along the different cartilage zones was reported, with the highest results shown at 84 days follow-up, but contrarily, the CT showed deep clefts and deep erosions of the AC, with an abnormal distribution of chondrocytes (Figures 5, 6). A bad basal integration, a lack of tidemark formation and SB abnormalities were reported in the CT group, but better results were obtained 84 days after the infiltration. Regarding the parameter inflammation, inflammatory infiltration was reported in the CT group both at 56- and 84-days follow-up, whereas little presence of inflammatory cells was showed in the TRT group. The presence of blood vessels was specially increased 56 days after infiltration in the TRT group, however, the same group showed the best score for this parameter at 84 days follow-up. Finally, regarding overall assessment, the repaired tissue in the TRT group had characteristics similar of those of normal hyaline cartilage, however, the defect in the CT group was regenerated with fibrocartilage-like tissue (Figure 7).

## 4. Discussion

In our study, better histological scores were obtained in rabbits treated with IA combined with IO injections of PRGF. The use of PRP to treat musculoskeletal disorders, including OA and chondral defects, has gained interest in veterinary clinical practice during the last years and IA infiltration has shown positive clinical and functional outcomes (43–47). However, in patients severely affected, the IA therapy is not able to reach all the structures involved in OA pathology, especially the SB (48).

Accordingly, several authors have proposed IA combined with IO infiltration of PRP to provide a more comprehensive treatment to patients severely affected, and previous results have already showed that this infiltration methodology is secure and provides better and longer functional and clinical improvements compared with IA injections alone (28, 34–37, 48–51). Moreover, our research team has recently reported that rabbits with acute full depth chondral defect treated with IA together with IO PRGF infiltrations showed better macroscopical AC repair than those treated with single IA injections of PRGF at 56- and 84-days follow-up (38).

The results obtained in the present research are in concordance with the ones previously published. The OARSI grading system uses 4 different parameters to assess the repair tissue and the defect gets a score ranging from 0 (normal AC), to 24 (extremely damaged AC) (41). For all studied parameters included in the OARSI score, rabbits treated with the combination of IA and IO injections of PRGF showed significantly lower score than those treated with IA-only PRGF injection both at 56- and 84 days follow up.

On the other hand, the ICRS II Histology Scoring System is composed by 13 parameters that should be evaluated based on the visual analog scale and graded from 0 (extremely damaged AC, with fibrous tissue appearance) to 100 (normal hyaline cartilage) (40). In the present study higher scores were obtained for all parameters in rabbits treated with both, IA and IO PRGF at 56- and 84-days follow-up. However, for the criteria “vascularity” at 56- days follow up, the rabbits in TRT group showed significantly lower score, thus meaning greater presence of blood vessels, compared to CT group. These results agree with the ones previously published by our research team, in which a higher macroscopical presence of blood vessels was reported in the IO treated rabbits (38). This angiogenic activity showed in the TRT group is mainly modulated by the presence of pro-angiogenic platelet-derived vascular endothelial growth factor within the PRGF, and other mediators such as transforming growth factor-β and fibroblast growth factor-2, which produce new blood vessels by stimulating endothelial cells (52–54). The angiogenesis process contributes to the healing of musculoskeletal disorders in areas of poor vascularization, such as chondral defects, meniscal tears, tendon injuries and other areas with poor vascularity (55).

Moreover, in our study, the TRT group showed significant lower presence of inflammatory cells than the CT group at both study points. This could be due to the anti-inflammatory nature of PRP. Several authors have demonstrated that platelets modulate inflammation, both *in vitro* and *in vivo* (56–58). Osterman et al. studied the effect of leukocyte-poor PRP and leukocyte-rich PRP *in vitro* and both preparations showed decreased expression of genes correlated with the inflammatory process in OA cartilage (56). In the same line, Lacko et al. investigated the effect of PRP injections in patients with knee OA and a significant decrease in pro-inflammatory makers was reported (57). Our results are also in concordance with the ones published by Muinos-Lopez et al. (59) that concluded that IO infiltration of PRP induced a greater reduction than IA infiltration in the population of monocytes and lymphocytes of the synovial fluid (59). Furthermore, Lychagin et al. assessed the inflammatory response of OA patients after IO infiltration of PRP by quantifying the serum oligomeric matrix protein. They concluded that, after IO infiltration, the biomarker level started to decrease (51).

Additionally, in our study, in the TRT group most of the surface of the repair tissue was integrated well with the surrounded healthy cartilage, while in the CT group, clefts in the superficial, transitional, and deep zones were reported. Furthermore, in some CT rabbits a complete loss of superficial, mid and deep zones of the AC were reported. In the TRT group, a normal hyaline cartilage morphology was achieved. Elongated cells were arranged parallel to the surface in the superficial zone, while in the mid/deep zone spherical cells were arranged forming columns. However, the repair tissue in the CT group was mainly fibrous and the presence of chondrocytes clusters and hypocellularity of chondrocytes in the mid/deep zone was significantly higher than in the TRT group. In terms of SB repair, a continuous layer of trabecular bone was well formed below the cartilage in the TRT group. Contrarily, in the CT group the SB was not well regenerated and large areas of structural disorganization and fibrocartilage were present. The obtained histological results could explain the clinical and functional improvements reported by some authors after IO infiltration of PRP in osteoarthritic human patients (34–37, 48).

SB lies subjacent to the deeper layer of AC and provides nutritional and mechanical support for the hyaline cartilage, indicating that microenvironmental changes in SB can affects cartilage metabolism (60). Recently, several studies elucidating bone-cartilage crosstalk and SB metabolism have reported that OA is not a cartilage-only pathology, but a whole-joint disorder (30). In general terms, it is believed that abnormal mechanical loads lead to an increase in SB turnover, accelerating bone remodeling beneath the tidemark. Finally, aberrant mechanical support due to SB sclerosis induces AC destruction (61–63). In our study, IO PRGF enhances SB healing and prevented OA progression. The IO infiltration with PRGF might enhance the performance of the SB by inducing the influx of additional MSCs, that help to maintain tissue homeostasis, thus diminishing the activation of proinflammatory cytokines preventing further cartilage loss. Moreover, Ganguly et al. (33) have recently investigated the changes in SB MSCs before and after combined IA and IO infiltrations of PRP in patients with hip OA. Their data showed that combined IO and IA infiltrations of this biological therapy, enhanced SB MSCs proliferative and stress-resistance capacities (33). This could be another reason why the IO infiltration of PRGF provides a greater regeneration of the SB and the AC in rabbits with full depth chondral defects.

Furthermore, the combination of IA and IO infiltration with PRGF seems to exhibit longer lasting effects than the IA infiltration alone. In the ORSI scale, significant better scores were achieved by the TRT group 84-days following infiltration compared to those obtained at 56-days follow-up for all studied parameters unless chondrocyte density. However, in the CT group, only the criterion articular cartilage surface showed a significant improvement at 84-days follow up. These results suggests that the IO PRGF still playing an important role in the regeneration process of full depth chondral defect 84-days after infiltration, however, the therapeutic potential of IA PRGF by this time seems limited.

Despite various studies have reported that IO infiltration of PRGF is a safe technique, this approach has some limitations. The procedure is invasive, and it should be performed in the surgery room, using fluoroscopy or ultrasounds. Sedation and general or local anesthesia are needed, which makes IO infiltration a more expensive technique than IA infiltration. To our knowledge, this research is the first one in this study field in which histological AC changes have been assessed after IO infiltration of PRGF. Even if positive outcomes have been reported with this new approach, further clinical studies are required in companion animals to evaluate clinical and biomechanical improvements.

## 5. Conclusions

The combination of IA and IO infiltration of PRGF provides a more comprehensive treatment of full depth chondral defects in severely affected patients. The treatment can reach all tissues implicated in OA pathogenesis, including the SB.

Better histological outcomes have been achieved in rabbits treated with the combination of IA and IO injections of PRGF than in the ones treated with IA-only PRGF injections. The benefits of IO infiltration of this therapy are durable and additionally, no clinical complications associated with the treatment have been reported. Although the results are positive, further studies in this field are necessary to assess the clinical and functional improvements that this new approach could contribute to veterinary practice.

## Data availability statement

The raw data supporting the conclusions of this article will be made available by the authors, without undue reservation.

## Ethics statement

The animal study was reviewed and approved by the Ethics Committee of Animal Welfare (CEEA) of the university CEU Cardenal Herrera of Valencia.

## Author contributions

Conceptualization: JS and JC. Methodology and visualization: MR and JS. Software: MR. Validation: MR, JS, ED, and JC. Formal analysis and writing – original draft preparation: MT-T. Investigation: MT-T, LM-P, AR, ED, and PP. Resources and project administration: JS. Data curation: MT-T, PP, and JC. Writing – review and editing: MT-T and ED. Supervision: ED and MR. Funding acquisition: JC. All authors contributed to the article and approved the submitted version.
